# Retraction: Relationship between microRNA-27a and efficacy of neoadjuvant chemotherapy in gastric cancer and its mechanism in gastric cancer cell growth and metastasis

**DOI:** 10.1042/BSR-2018-1175_RET

**Published:** 2021-08-06

**Keywords:** Cell growth, Gastric cancer, Metastasis, MicroRNA-27a, Neoadjuvant chemotherapy

This Retraction follows an Expression of Concern relating to this article previously published by Portland Press.

This article is being retracted from *Bioscience Reports* at the request of the Editor-in-Chief and the Editorial Board following receipt of a notification from a reader, alerting the Editorial Board to duplicated regions and images in Figures 4D, 4E, 4F, 5C, 5D, 5E, 5F, 7C, 7D, 7E and 7F.

The authors have been contacted in regards to the Retraction, and have not responded to the Journal's queries and the concerns raised. Given the extent of the issues raised, the Editorial Board stand by the decision to retract the article.

